# Variable Susceptibility to Gallium Compounds of Major Cystic Fibrosis Pathogens

**DOI:** 10.1021/acsinfecdis.1c00409

**Published:** 2021-12-29

**Authors:** Daniela Visaggio, Emanuela Frangipani, Sarah Hijazi, Mattia Pirolo, Livia Leoni, Giordano Rampioni, Francesco Imperi, Lawrence Bernstein, Raffaella Sorrentino, Francesca Ungaro, Paolo Visca

**Affiliations:** † Department of Science, Roma Tre University, 00146 Rome, Italy; ‡ Santa Lucia Fundation IRCCS, 00179 Rome, Italy; § Department of Biomolecular Sciences, University of Urbino Carlo Bo, 61029 Urbino, Italy; ∥ Gallixa, Menlo Park, 94025 California, United States; ⊥ Department of Molecular Medicine and Medical Biotechnology, School of Medicine, University of Naples Federico II, 80138 Naples, Italy; # Department of Pharmacy, University of Naples Federico II, 80131 Naples, Italy

**Keywords:** antimicrobial susceptibility testing, cystic fibrosis, gallium maltolate, gallium nitrate, gallium protoporphyrin IX

## Abstract

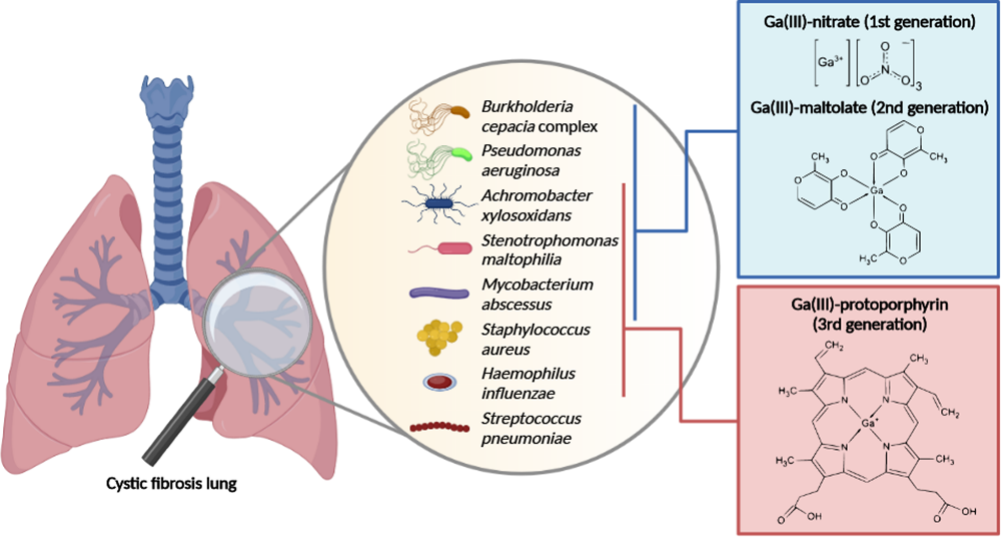

The decreasing efficacy of existing antibiotics against pulmonary pathogens that affect cystic fibrosis (CF) patients calls for the development of novel antimicrobials. Iron uptake and metabolism are vital processes for bacteria, hence potential therapeutic targets. Gallium [Ga(III)] is a ferric iron-mimetic that inhibits bacterial growth by disrupting iron uptake and metabolism. In this work we evaluate the efficacy of three Ga(III) compounds, namely, Ga(NO_3_)_3_, (GaN), Ga(III)-maltolate (GaM), and Ga(III)-protoporphyrin IX (GaPPIX), against a collection of CF pathogens using both reference media and media mimicking biological fluids. All CF pathogens, except *Streptococcus pneumoniae*, were susceptible to at least one Ga(III) compound. Notably, *Mycobacterium abscessus* and *Stenotrophomonas maltophilia* were susceptible to all Ga(III) compounds. *Achromobacter xylosoxidans*, *Burkholderia cepacia* complex, and *Pseudomonas aeruginosa* were more susceptible to GaN and GaM, whereas *Staphylococcus aureus* and *Haemophilus influenzae* were more sensitive to GaPPIX. The results of this study support the development of Ga(III)-based therapy as a broad-spectrum strategy to treat CF lung infections.

The declining efficacy of existing antibiotics against many bacterial pathogens urgently calls for the development of novel antibacterial therapies.^1^ A promising strategy to fight bacterial infections is to exploit pathogens’ nutritional vulnerability by impairing the acquisition of essential nutrients, e.g., iron. Indeed, iron is a key nutrient for bacterial pathogens, being a cofactor for many enzymes involved in critical metabolic and reproductive functions.^2^ During infection, iron bioavailability is extremely low due to its sequestration by the host, which imposes a nutritional stress on invading pathogens as part of the innate immune response.^3^ The post-transition, iron-mimetic metal gallium [Ga(III)], the active component of the FDA-approved citrated gallium nitrate, has successfully been repurposed as an antimicrobial agent.^4,5^ Unlike Fe(III), Ga(III) is not reducible under physiological conditions and thus cannot take part in redox reactions, preventing a number of iron-dependent essential functions.^6,7^ Fourteen years after the seminal study unraveling the anti-*Pseudomonas* activity of Ga(III),^8^ the possibility of using Ga(III) to combat bacterial infections is becoming a viable opportunity. The well-documented in vitro antibacterial activity of Ga(III), as well as its promising protective effect against pathogens in mouse models of infections,^9^ combined with favorable pharmacological properties^10^ have paved the way for clinical trials evaluating the pharmacokinetics, safety, tolerability and efficacy of Ga(III) as an antibacterial agent.

Phase 1 and 2 clinical trials (NCT01093521 and NCT02354859, respectively) demonstrated that Ga(III) is safe, well tolerated, does not induce significant nephrotoxicity, and is able to improve lung function in cystic fibrosis (CF) patients suffering from *Pseudomonas aeruginosa* chronic lung infection.^11^ No data are yet available concerning the ongoing clinical trials (ABATE, phase 1/2 NCT04294043 and NCT03669614), which aim to evaluate the efficacy in CF patients of intravenously administered Ga(III) against nontuberculous *Mycobacterium* species, and of an inhaled Ga(III) formulation (AR-501) against *P. aeruginosa*, respectively.

All clinical trials to date using Ga(III) as an antibacterial agent have been designed for patients suffering from CF, an autosomal recessive genetic disorder characterized by mutations of the CF transmembrane conductance regulator (CFTR) gene. Mutations in CFTR lead to a multifactorial syndrome, with pulmonary manifestations representing the major contributor to morbidity and mortality.^12^ The progressive decline in lung function is caused primarily by bacterial infections, inflammation, and consequent tissue damage. Although antibiotics have prolonged the longevity of CF patients, they have also selected for resistance,^13^ making the eradication of established infection problematic.^14^

While *P. aeruginosa* remains the dominant infectious agent in CF, other opportunistic pathogens such as *Achromobacter xylosoxidans*, *Burkholderia cepacia* complex (Bcc), and *Stenotrophomonas maltophilia*, which are infrequently responsible for disease in healthy hosts, may cause both acute and chronic infection of CF airways, often associated with poor outcomes.^15^ Moreover, CF patients may also suffer from polymicrobial lung infections, which are even harder to treat than monomicrobial ones. Indeed, coinfection by *Staphylococcus aureus* and *P. aeruginosa* is associated with worse respiratory function compared to infection by *S. aureus* only.^16,17^ Moreover, mainly during childhood, *S. aureus* can also coexist in the CF lung with *Haemophilus influenzae*.^17^

Since all these pathogens require iron for proliferating in vivo, Ga(III) may be effective as a broad-spectrum antibacterial to treat CF lung infections. With this perspective, we investigated the antibacterial effects of three different Ga(III)-based compounds on a representative collection of CF pathogens belonging to 10 different species (Table 1 and Table S1). The selected compounds belong to the first, second, and third generations of Ga(III) formulations, respectively: Ga(III)-nitrate (GaN), Ga(III)-maltolate (GaM), and Ga(III)-protoporphyrin IX (GaPPIX).^18^ The rationale for using different Ga(III) complexes relies on the observation that several pathways for the uptake of Fe(III) complexes exist in bacterial pathogens, some of which are highly conserved and widely employed, while others are unique to individual species.^19^ The collection of CF pathogens includes, for each species, one reference strain and one or more clinical isolates, mainly from CF lung infections (Table S1).

**Table 1:** MIC (μM) of Ga(III) Compounds for Different Strain of Major CF Pathogens^a^

	CAMHB/HTM^b^/THYB^c^			ID-CAMHB/DHTM^b^/DTHYB^c^			ASM			RPMI-HS-CAA^d^		
bacterial strain	GaN	GaM	GaPPIX	GaN	GaM	GaPPIX	GaN	GaM	GaPPIX	GaN	GaM	GaPPIX
*Achromobacter xylosoxidans* ATCC 27061^T^	>128	>128	>128	>128	>128	**32**	>128	>128	**≤0.0075**	ND	ND	ND
*A. xylosoxidans* CF-2	>128	>128	>128	>128	>128	**32**	>128	>128	**0.12**	ND	ND	ND
*A. xylosoxidans* CF-3	>128	>128	>128	>128	>128	**32**	>128	>128	**0.12**	**32**	**16**	64
*A. xylosoxidans* CF-4	>128	>128	>128	>128	>128	**16**	>128	>128	**≤0.0075**	**8**	**4**	**2**
*Burkholderia cenocepacia* LMG 16656^T^	>128	>128	>128	>128	>128	>128	>128	>128	**32**	**1**	**1**	**32**
*B. cenocepacia* FFC 0076	>128	>128	>128	>128	>128	>128	>128	>128	**4**	**2**	**2**	64
*Burkholderia dolosa* LMG 18943T	>128	>128	>128	>128	>128	>128	>128	>128	>128	**2**	**2**	128
*B. dolosa* FFC 0305	>128	>128	>128	>128	>128	>128	>128	>128	>128	**4**	**4**	128
*Burkholderia multivorans* LMG 31010T	>128	>128	>128	>128	>128	>128	>128	>128	>128	**4**	**2**	128
*B. multivorans* 454	>128	>128	>128	>128	>128	>128	>128	>128	>128	**4**	**2**	128
*Haemophilus influenzae* ATCC 49247^b^	>128	>128	**≤0.0075**	>128	>128	**≤0.0075**	ND	ND	ND	>128	>128	**0.5**
*H. influenzae* ATCC 9833	>128	>128	**≤0.0075**	64	>128	**≤0.0075**	ND	ND	ND	>128	>128	**0.5**
*H. influenzae* FC 89	>128	>128	**≤0.0075**	>128	>128	**≤0.0075**	ND	ND	ND	>128	>128	**2**
*H. influenzae* FC 104	>128	>128	**≤0.0075**	>128	>128	**≤0.0075**	ND	ND	ND	>128	>128	**2**
*Mycobacterium abscessus* ATCC 19977^T^	>128	128	**1**	>128	128	**0.06**	>128	**32**	**4**	**4**	**4**	**16**
*M. abscessus* ISS6	>128	128	**0.25**	>128	128	**0.12**	>128	**32**	**0.5**	**2**	**2**	**8**
*M. abscessus* ISS7	>128	128	**1**	>128	128	**0.06**	>128	**32**	**8**	**4**	**4**	64
*M. abscessus* ISS9	>128	128	**1**	>128	128	**0.5**	>128	**32**	**8**	**8**	**8**	64
*Pseudomonas aeruginosa* PAO1 (ATCC 15692)	>128	>128	>128	64	>128	>128	**16**	**8**	128	**8**	**8**	>128
*P. aeruginosa* TR1	128	>128	>128	64	>128	>128	**16**	**16**	**0.012**	**2**	**2**	**4**
*P. aeruginosa* FM12	>128	>128	>128	64	>128	>128	**16**	**16**	>128	**16**	**8**	>128
*P. aeruginosa* FM13	>128	>128	>128	64	>128	>128	**16**	**16**	**16**	**8**	**4**	64
*Staphylococcus aureus* ATCC 25923	>128	>128	**0.12**	>128	>128	**0.12**	>128	>128	**2**	>128	>128	>128
*S. aureus* ATCC 43300	>128	>128	**0.06**	>128	>128	**0.06**	>128	128	**2**	128	>128	>128
*S. aureus* BG-1	>128	>128	**0.06**	>128	>128	**0.06**	>128	128	**1**	128	64	>128
*S. aureus* BG-6	>128	>128	**0.06**	>128	>128	**0.03**	>128	64	**0.25**	64	128	>128
*Stenotrophomonas maltophilia* ATCC 13637^T^	>128	>128	128	>128	>128	64	>128	>128	**8**	**8**	**8**	**0.25**
*S. maltophilia* K279a	>128	>128	>128	>128	>128	128	>128	>128	**4**	**4**	**4**	**0.06**
*S. maltophilia* OBGTC23	>128	>128	>128	>128	>128	64	>128	>128	**0.03**	**0.25**	**0.25**	**0.03**
*S. maltophilia* OBGTC26	>128	>128	>128	>128	>128	64	>128	>128	**0.03**	**4**	**4**	**0.12**
*Streptococcus pneumoniae* ATCC 33400^T^	>128	>128	>128	>128	>128	128	ND	ND	ND	>128	128	>128
*S. pneumoniae* PFC-01	>128	>128	128	>128	>128	64	ND	ND	ND	>128	64	64
*S. pneumoniae* PFC-02	>128	>128	128	>128	>128	64	ND	ND	ND	>128	64	>128
*S. pneumoniae* PFC-04	>128	>128	64	>128	>128	64	ND	ND	ND	>128	64	64

a Abbreviations: ^T^, type strain; ND, not determined due to poor growth.

b HTM/DHTM supplemented with 0.063 μM and 0.125 μM PPIX, respectively, were used to allow *H. influenzae* growth.

c THYB/DHTYB were used only for *S. pneumoniae*.

d Only in the case of *H. influenzae* and *S. pneumoniae*, was RPMI-HS-CAA supplemented with 3.3 μg/mL hypoxanthine, 100 μg/mL l-alanine, 55 μg/mL l-cysteine hydrochloride, 6.6 μg/mL NAD, and 8 μg/mL uracil, to allow bacterial growth. Arbitrarily setting the resistance breakpoint at MIC >32 μM, the MIC values for susceptible isolates are shown in bold type. For each strain/condition, susceptibility tests were performed at least in triplicate, yielding the same MIC results.

Since Ga(III) is a metabolic competitor of Fe(III), its antibacterial activity depends on the iron concentration of the test medium, being enhanced under conditions of iron scarcity.^5^ Therefore, in addition to the reference iron-rich medium recommended for antibacterial-susceptibility testing (cation-adjusted Mueller Hinton Broth; CAMHB),^20^ also the iron-depleted CAMHB (ID-CAMHB)^21^ was used. In addition, to mimic the environment encountered by pulmonary pathogens during infection, the artificial sputum medium (ASM)^22^ as well as the chemically defined tissue culture medium RPMI 1640 supplemented with 10% human serum (HS) and 0.5% casamino acids (RPMI-HS-CAA), were employed. Given the heterogeneity of nutritional requirement of CF pathogens, the ability to grow in the selected media was preliminarily assessed for the reference strains of each species.

All the above media supported the growth of nonfastidious species except the *A. xylosoxidans* ATCC 2761 type strain, which failed to grow in RPMI-HS-CAA (Figure S1).

Concerning the fastidious species, *H. influenzae* lacks all enzymes for biosynthesis of the porphyrin ring^23^ but has evolved redundant mechanisms to obtain heme (i.e., Hgps, Hup, HxuCBA) from host hemoproteins, such as hemoglobin, hemoglobin-haptoglobin, myoglobin-haptoglobin, heme-hemopexin, heme-albumin, and catalase.^23−26^ In addition, *H. influenzae* possesses a ferrochelatase which reversibly inserts iron into PPIX to form heme, so that it can grow when fed with PPIX and an iron source such as ferri-transferrin.^27^ Therefore, to avoid an excess of iron, *H. influenzae* was grown in HTM medium supplemented with PPIX instead of hemin. To determine the optimal PPIX concentration, the growth of *H. influenzae* ATCC 49247^T^ was compared in hemin-free HTM and in the same medium treated with Chelex to reduce iron content (DHTM), both supplemented with increasing PPIX concentrations. Results showed that the presence of PPIX stimulated *H. influenzae* growth, both in HTM and in DHTM, in a dose-dependent manner (Figure 1A). Moreover, *H. influenzae* growth reached a plateau at PPIX concentrations ≥0.063 and ≥0.125 μM in HTM and DHTM, respectively (Figure 1A), and no further increase in growth yield was observed for PPIX concentrations up to 23 μM (data not shown). Therefore, Ga(III)-susceptibility tests on *H. influenzae* were conducted in HTM and DHTM in the presence of 0.063 and 0.125 μM PPIX, respectively.

**Figure 1:**
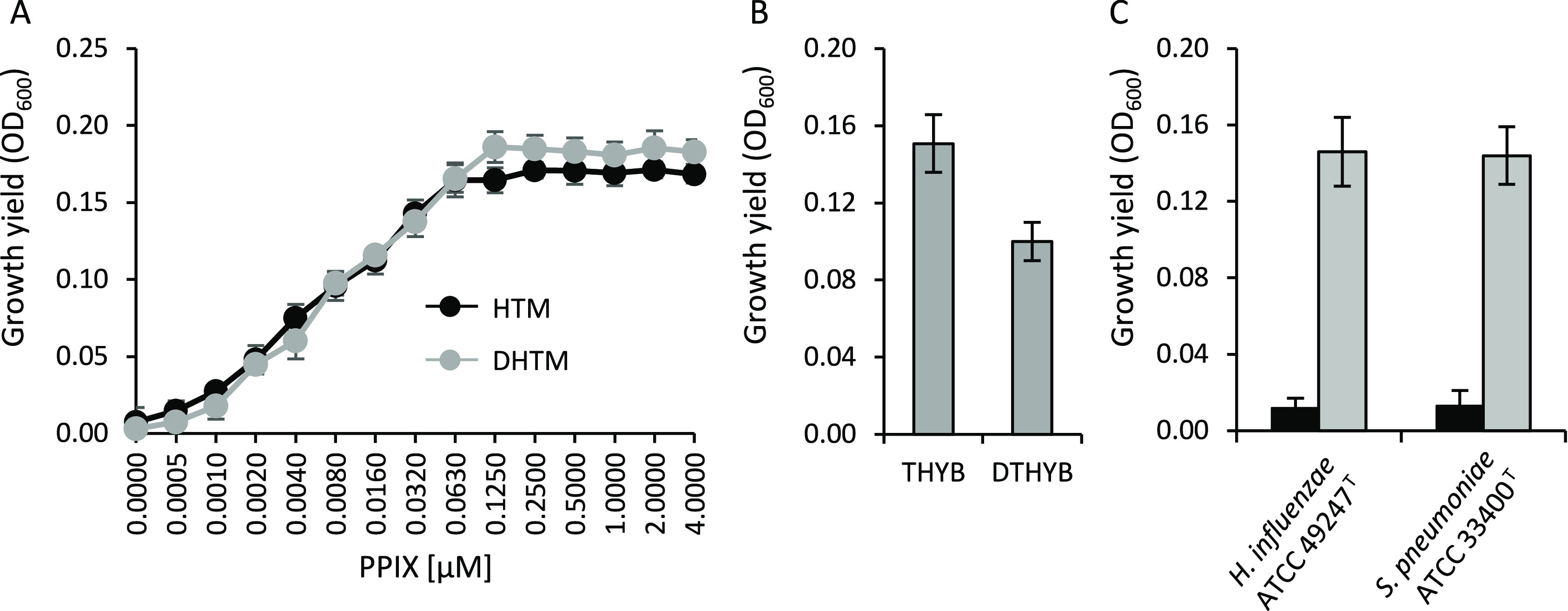
Growth of *H. influenzae* and *S. pneumoniae* in different media for antimicrobial susceptibility testing. (A) Growth yields of *H. influenzae* ATCC 49247^T^ in HTM or DHTM in the presence of increasing concentrations of PPIX (from 0.0005 to 4 μM). (B) Growth yields of *S. pneumoniae* ATCC 33400^T^ in THYB or DTHYB. (C) Growth yields of *S. pneumoniae* ATCC 33400^T^ and *H. influenzae* ATCC 49247^T^ in RPMI-HS-CAA (black bars), or RPMI-HS-CAA with supplements (3.3 μg/mL hypoxanthine, 100 μg/mL l-alanine, 55 μg/mL l-cysteine hydrochloride, 6.6 μg/mL NAD, and 8 μg/mL uracil), (gray bars). Bacteria were inoculated (ca. 5 × 10^5^ CFU/mL) into 96-well microtiter plates. The OD_600_ was determined after 24 h incubation at 37 °C. *S. pneumoniae* ATCC 33400^T^ was grown in an atmosphere containing 5% CO_2_. Data are the means of triplicate experiments ± standard deviation.

To test the activity of Ga(III) compounds on *S. pneumoniae*, Todd–Hewitt Broth supplemented with 0.5% of yeast extract (THYB)^28^ and its Chelex-treated iron-depleted derivative (DTHYB)^29^ were used. *S. pneumoniae* ATCC 33400^T^ was able to grow in both media, although slightly less in DTHYB compared to THYB (Figure 1B), likely due to iron scarcity.

Both *H. influenzae* and *S. pneumoniae* did not grow in ASM and in RPMI-HS-CAA, likely due to the absence of essential factors strictly required for the proliferation of these pathogens. Since it is known that the growth of *H. influenzae* in RPMI-1640 requires the addition of supplements, namely, 3.3 μg/mL hypoxanthine, 100 μg/mL l-alanine, 55 μg/mL l-cysteine hydrochloride, 6.6 μg/mL NAD, and 8 μg/mL uracil,^30^ the ability of fastidious bacteria to grow in RPMI-HS-CAA supplemented with the above factors was investigated. The addition of supplements allowed the growth of both *H. influenzae* and *S. pneumoniae* type strains (Figure 1C), and the supplemented RPMI-HS-CAA medium was therefore used for subsequent Ga(III) susceptibility tests in these strains. Since Ga(III) antibacterial activity is counteracted by iron, the total iron concentration in the test media was determined by inductively coupled plasma optical emission spectrometry (ICP-OES). As expected, the treatment of CAMHB, HTM, and THYB with the Chelex resin strongly lowered their iron concentration (Table S2). Moreover, the iron concentration of ASM was comparable to that of the CAMHB rich medium (3.305 μM in CAMHB and 3.880 μM in ASM), and higher than that of RPMI-HS-CAA (1.820 μM). Since the iron concentration in RPMI-CAA is only 0.210 μM, it can be argued that HS supplies RPMI-CAA with significant iron levels (Table S2).

After having established appropriate culture conditions for Ga(III) susceptibility testing of CF pathogens, the MIC of different Ga(III) compounds was determined. Since the peak serum concentration of Ga(III) achievable during intravenous human therapy is ca. 30 μM,^6,31^ we arbitrarily set the resistance breakpoint at MIC ≥ 32 μM. Interestingly, the MIC of Ga(III) compounds greatly varied depending on the test medium, suggesting that both iron content and nutrient composition affect the outcome of Ga(III) susceptibility assays. In CAMHB, HTM, and THYB and in their iron-depleted formulations (ID-CAMHB, DHTM, and DTHYB), all strains were resistant to GaN and GaM, while the majority of them, namely, two clinical *A. xylosoxidans* isolates, Bcc, *Mycobacterium abscessus*, *P. aeruginosa*, and *S. maltophilia*, were susceptible to both GaM and GaN in RPMI-HS-CAA (Table 1). It is plausible that the extreme iron limitation due to the presence of iron-binding proteins in HS can improve the antibacterial activity of Ga(III) in RPMI-HS-CAA with respect to other media.^32^

The MIC of GaN in RPMI-HS-CAA was identical or only twice that of GaM for all the susceptible species, implying that the inhibitory activity of these compounds is nearly equivalent in vitro. In ASM, GaM was effective on both *P. aeruginosa* and *M. abscessus*, whereas only *P. aeruginosa* was susceptible to GaN. While the inhibitory activity of both GaM and GaN were previously documented for both *P. aeruginosa* and *M. abscessus*,^32−34^ unexpected results were obtained with bacteria belonging to the Bcc. Indeed, previous work argued against an antibacterial effect of GaN on Bcc, since high concentrations of GaN (from 4 to 18 mg/L, corresponding to 30–260 μM) were required to inhibit the growth of Bcc in a chemically defined (minimal salt) medium.^35^ However, in the present work, lower concentrations of GaM and GaN (1–4 μM) prevented Bcc growth in RPMI-HS-CAA (Table 1). This apparent discrepancy highlights the need to establish appropriate methods to assess the antibacterial activity of Ga(III) compounds, depending on the test species. The anti-Bcc activity of Ga(III) in the presence of HS is of particular relevance considering that septicemia often occurs when Bcc pulmonary infections evolve into a potentially fatal condition known as “cepacia syndrome”.^36^ This raises the possibility that intravenous administration of GaN, which is already approved by the FDA, could help in treatment of systemic Bcc infection. Similar to Bcc, the previously established MIC of GaN for *S. maltophilia* (MIC = 512 μg/mL corresponding to 2000 μM)^37^ was higher than that determined in this study using RPMI-HS-CAA (MIC = 0.25–8 μM), and also for this species it should be taken into account that the previous MIC assays were performed in CAMHB,^37^ where the elevated iron content would mask Ga(III) activity (Table 1; Table S2). Interestingly, also *A. xylosoxidans* CF-3 and CF-4 were susceptible to GaN and GaM in RPMI-HS-CAA, providing the first evidence of Ga(III) activity against this CF pathogen. *H. influenzae, S. aureus*, and *S. pneumoniae* were completely resistant to both GaN and GaM in all media used in the present study. In addition to medium composition and iron levels, species- and strain-specific factors could affect Ga(III) activity against CF pathogens. For instance, in *P. aeruginosa* the pyoverdine siderophore and exoproteases increase Ga(III) tolerance in media supplemented with iron chelating proteins,^38^ whereas the pyochelin siderophore decreases Ga(III) tolerance by contributing to its internalization.^39^ It can therefore be speculated that siderophores, exoproteases, and possibly other species- and strain-specific factors could explain the variable response to Ga(III) compounds in other CF pathogens.

Intriguingly, the susceptibility profile of CF pathogens to the heme mimetic GaPPIX was completely different from that of GaN and GaM. All CF pathogens, except *B. dolosa*, *B. multivorans*, and *S. pneumoniae*, were susceptible to GaPPIX in at least one of the media used in this study (Table 1). Intriguingly, *H. influenzae*, *S. aureus*, and to a lesser extent, also *M. abscessus* were extremely susceptible to GaPPIX even in the iron rich media (CAMHB and HTM; Table 1; Table S2). Moreover, for *S. aureus* and *H. influenzae* no differences in GaPPIX MIC were observed between iron-rich and iron-deprived media (CAMHB/HTM and ID-CAMHB/DHTM), denoting that the mechanism of action of GaPPIX in these species is not correlated to the iron starvation status of bacterial cells.

Contrarily, *A. xylosoxidans*, which was resistant to GaPPIX in CAMHB, became susceptible in ID-CAMHB (MIC = 16–32 μM). Differences in the MIC values between the CAMHB and ID-CAMHB were also evident in *M. abscessus* (MIC = 0.25–1 μM in CAMHB and MIC = 0.06–0.5 μM in ID-CAMHB, Table 1). Therefore, unlike in *S. aureus* and *H. influenzae*, iron interferes with GaPPIX activity on *A. xylosoxidans* and *M. abscessus*.

Factors other than iron availability may influence the bacterial susceptibility to GaPPIX in media that mimic body fluids such as RPMI-HS-CAA and ASM. In particular, *A. xylosoxidans*, *B. cenocepacia*, *M. abscessus*, two *P. aeruginosa* strains (TR1 and FM13), *S. aureus*, and *S. malthophilia* were found to be susceptible to GaPPIX in ASM (Table 1). Except for two *S. malthophilia* strains, which displayed lower GaPPIX MIC in RPMI-HS-CAA than in ASM, the opposite was true for *M. abscessus*, *A. xylosoxidans*, some strains of *P. aeruginosa*, Bcc, and *S. aureus* (Table 1).

The ineffectiveness of GaPPIX in suppressing *S. aureus* growth in RPMI-HS-CAA is in line with previous results showing that human serum albumin (HSA) binds GaPPIX and suppressing its antibacterial activity.^32^ To verify a similar effect in *M. abscessus*, the MIC of GaPPIX was determined in CAMHB and in RPMI-CAA (without HS) supplemented or not with bovine serum albumin (BSA), a protein sharing 76% sequence identity and the same heme-binding properties as HSA.^40^ Addition of 5 mg/mL BSA to RPMI-CAA (equaling the final concentration of HSA in RPMI-HS) dramatically increased the MIC of GaPPIX in *M. abscessus* (Table 2). A similar effect was also observed upon addition of BSA to CAMHB (Table 2).

**Table 2:** Effect of BSA on the MIC (μM) of GaPPIX for *M. abscessus*^a^

	CAMHB		RPMI-CAA	
bacterial strain	no BSA	5 mg/mL BSA	no BSA	5 mg/mL BSA
*Mycobacterium abscessus* ATCC 19977^T^	1	16	≤0.12	4
*M. abscessus* ISS6	0.25	2	≤0.12	16
*M. abscessus* ISS7	1	128	≤0.12	128
*M. abscessus* ISS9	1	128	≤0.12	128

a Abbreviations: ^T^, type strain. For each strain/condition, susceptibility tests were performed at least in triplicate, yielding the same MIC results.

A marked increase in the GaPPIX MIC in RPMI-HS-CAA was also observed for *H. influenzae*, likely due to heme acquisition from hemoglobin, hemopexin, albumin, and catalase provided by HS.^23^

Altogether, our results indicate that albumin and probably other serum components, besides iron, can interfere with GaPPIX antibacterial activity, and this should be taken into account in preclinical testing of this compound.

Although the activity of GaPPIX on *S. aureus* and *M. abscessus* was expected based on previous reports,^32,33^ here novel evidence of GaPPIX efficacy on minor CF pathogens, namely *H. influenzae* and the Gram-negative non fermenting bacteria *A. xylosoxidans* and *S. malthopilia*, is provided for the first time.

It is well-known that GaPPIX exploits heme uptake systems to enter bacterial cells,^41,42^ therefore the number, type and level of expression of heme-utilization systems are likely to account for the variable susceptibility to GaPPIX.^32,42^

While heme uptake systems are extensively characterized in *H. influenzae*, little it is known about the heme acquisition pathways of *A. xylosoxidans* and *S. malthophilia*. However, genome analysis of both environmental and clinical *S. malthophilia* strains revealed the presence of several genes putatively involved in heme uptake,^43^ which can explain the susceptibility of this species to GaPPIX. No information is so far available about heme acquisition by *A. xylosoxidans*.

In conclusion, the strong species-specific inhibitory activity of some Ga(III) compounds against individual CF pathogens could pave the way for future development of Ga(III)-based antibacterial therapies in CF patients. Ga(III)-based compounds could be suitable for the setting up of patient-specific treatments based on the infecting species and the phase of infection. For instance, GaPPIX holds promise for the treatment of *H. influenzae* and *S. aureus* infections in children with CF, since these infections cause airways lesions and predispose to *P. aeruginosa* colonization.^17^ In adulthood, when CF airways are primarily colonized by *P. aeruginosa*, GaN and GaM are more likely to be effective. Of note, a recent study has demonstrated that the combination of GaPPIX with GaN displays substantial synergism against several bacteria species, including *P. aeruginosa* and *S. aureus*.^44^ Considering that CF patients suffer from polymicrobial infections in which different species coexist, the treatment with a combination of GaPPIX and GaN could hopefully alleviate or suppress the infection, delay the use of antibiotics and, consequently, the emergence of antibiotic resistance in CF pathogens. The potential of Ga(III) in combination with antibiotics should also be taken into account, since a synergistic activity of both GaN and GaPPIX with colistin and of GaN with piperacillin/tazobactam was recently documented.^11,44^ Although studies on *P. aeruginosa* have demonstrated that Ga(III) resistant mutants can emerge,^45,46^ resistance rates were found to be comparable or even lower than those of currently used antibiotics.^11^ However, the viable expectation of moving Ga(III)-based antimicrobials to the clinic poses the need for standardization of susceptibility testing procedures and guidelines, specifically designed for individual CF pathogens.

## Methods

### Bacterial Strains and Culture Conditions

Bacterial strains used in this work are listed in Table S1. All CF strains, except fastidious pathogens (*H. influenzae* and *S. pneumoniae*) and *M. abscessus*, were routinely cultured for 18 h in Tryptic Soy Broth (TSB, Acumedia) with vigorous shaking at 37 °C. *H. influenzae* and *S. pneumoniae* were cultured for 20 h in *Haemophilus* Test Medium (HTM) supplemented with 15 g/L agar (Acumedia) (HTMA), and Columbia agar supplemented with 5% sheep blood (Biomérieux), respectively. *S. pneumoniae* isolates were incubated in the presence of 5% CO_2_. *M. abscessus* was cultured for 72 h in TSB supplemented with 0.05% Tween 80 (Sigma-Aldrich). Bovine serum albumin (BSA, Sigma-Aldrich) was freshly prepared and added to the media at the final concentration of 5 mg/mL. When needed, 10 mM PPIX stock solution was freshly prepared in 10 mM NaOH.

### Iron Content Measurement

The iron concentration of all test media was determined by ICP-OES using an ICP-OES 710 Varian Spectrometer (Agilent Technologies). Briefly, the medium was supplemented with 5% HNO_3_, heated for 1 h at 90 °C, and filtered through a Millipore membrane (pore size 0.45 μm) prior to ICP-OES analysis.

### Test Media and Ga(III) Compounds

Appropriate media for individual CF pathogens were used for Ga(III)-susceptibility testing, namely: (i) CAMHB (Becton Dickinson); (ii) ID-CAMHB;^21^ (iii) HTM, depleted of heme; (iv) DHTM, prepared by treating HTM with 100 g/L of the metal-chelating Chelex 100 resin (Bio-Rad); (v) Todd–Hewitt Broth (Oxoid) supplemented with 0.5% yeast extract (THYB);^28^ (vi) DTHYB;^29^ (vii) RPMI-HS,^32^ supplemented with 0.5% CAA (Becton Dickinson) and/or *Hemophilus*-specific supplements [i.e., 3.3 μg/mL hypoxanthine (Nutritional Biochemicals Corporation), 100 μg/mL l-alanine (Merck), 55 μg/mL l-cysteine hydrochloride (Merck), 6.6 μg/mL nicotinamide adenine dinucleotide (NAD, Boehringer Mannheim GmbH), and 8 μg/mL uracil (Sigma)], and (viii) ASM,^22^ but replacing the mix of 20 amino acids with 5 g/L CAA.^47^

Three Ga(III) compounds were used in this study: (i) GaN [Ga(NO_3_)_3_·H_2_O, (Sigma-Aldrich)], freshly prepared as a 100 mM stock solution in water; (ii) GaM (NORAC Pharma), freshly prepared as a 22 mM stock solution in water; and (iii) GaPPIX (Frontier Scientific), prepared as a 25 mM stock solution in dimethyl sulfoxide (DMSO), and stored at 4 °C in the dark.

### Susceptibility Testing of Ga(III) Compounds

The antibacterial activity of Ga(III) compounds on CF pathogens was conducted using the microdilution method,^20^ with minor modifications. Briefly, bacteria were grown as outlined above and then transferred (ca. 5 × 10^5^ CFU/mL) in 200 μL of CAMHB/ID-CAMHB, HTM/DHTM, THYB/DTHYB, RPMI-HS-CAA, and ASM, in the presence of increasing concentrations (0–128 μM) of each Ga(III) compound (GaN, GaM, and GaPPIX), using 96-well microtiter plates. Since GaPPIX was dissolved in DMSO, for each bacterial strain control tests were performed with DMSO alone at the same concentrations used for GaPPIX susceptibility testing. All plates were incubated at 37 °C. *S. pneumoniae* was tested in 5% CO_2_ atmosphere. The MIC of Ga(III) compounds was defined as the lowest concentration that completely inhibited bacterial growth, as detected by the unaided eye.^20^ For all strains tested, except *A. xylosoxidans*, *S. maltophilia*, and *M. abscessus*, the MIC values were determined after 24 h incubation at 37 °C. For *A. xylosoxidans* and *S. maltophilia*, the MIC values in RPMI-HS-CAA were determined after 48 h incubation at 37 °C, due to poor growth after 24 h. For *M. abscessus* the MIC values were determined after 72 h incubation at 37 °C.

### Ethics Statement

This study does not involve patients but clinical isolates that were anonymously collected by different hospitals and/or research centers. Isolates were randomly selected from preexisting strain collections, which does not need ethical approval.

## Supporting Information Available

Table S1, list of bacterial strains used in this study; Table S2, iron concentrations; Figure S1, growth of CF reference strains in CAMHB, ID-CAMHB, RPMI-HS-CAA, and ASM (PDF)

## Abbreviations

CF: Cystic fibrosis
CFTR: Cystic fibrosis transmembrane conductance regulator
Bcc: *Burkholderia cepacia* complex
Ga(III): gallium
GaN: Ga(III)-nitrate
GaM: Ga(III)-maltolate
GaPPIX: Ga(III)-protoporphyrin IX
CAMHB: cation-adjusted Mueller Hinton Broth
ID-CAMHB: iron-depleted cation-adjusted Mueller–Hinton broth
CLSI: Clinical and Laboratory Standard Institute
PPIX: protoporphyrin IX
THBY: Todd–Hewitt Broth supplemented with 0.5% of yeast extract
DTHBY: iron-depleted Todd–Hewitt Broth supplemented with 0.5% of yeast extract
ICP-OES: inductively coupled plasma optical emission spectrometry
MIC: minimum inhibitory concentration
ASM: artificial sputum medium
RPMI-HS: RPMI 1640 supplemented with 10% of human serum
HS: human serum
RPMI-HS-CAA: RPMI-HS supplemented with 0.5% casamino acids
BSA: bovine serum albumin
HSA: human serum albumin
