# Supplementary material for: Variable Susceptibility to Gallium Compounds of Major Cystic Fibrosis Pathogens
Source: ACS Infect Dis. 2021 Dec 29;8(1):78–85. doi: 10.1021/acsinfecdis.1c00409 (PMC8762661; doi:10.1021/acsinfecdis.1c00409)
Supplement: Supplementary file 1 — id1c00409_si_001.pdf [file id1c00409_si_001.pdf]

SUPPORTING INFORMATION

Variable susceptibility to gallium compounds of major cystic fibrosis pathogens

Daniela Visaggio[a,b*], Emanuela Frangipani[c], Sarah Hijazi[a], Mattia Pirolo[a], Livia Leoni[a], Giordano Rampioni[a,b], Francesco Imperi[a,b], Lawrence Bernstein[d], Raffaella Sorrentino[e], Francesca Ungaro[f], Paolo Visca[a,b*]

[a]Department of Science, Roma Tre University, 00146 Rome, Italy

[b]Santa Lucia Fundation IRCCS, 00179 Rome, Italy

[c]Department of Biomolecular Sciences, University of Urbino Carlo Bo, 61029 Urbino, Italy

[d]Gallixa, Menlo Park, 94025 California, USA

[e]Department of Molecular Medicine and Medical Biotechnology, School of Medicine, University of Naples Federico II, 80138 Naples, Italy

[f]Department of Pharmacy, University of Naples Federico II, 80131 Naples, Italy

[*]Corresponding Authors: Paolo Visca, paolo.visca@uniroma3.it; Department of Science, Roma Tre University, Viale Guglielmo Marconi 446, 00146 Rome, Italy; Phone: +39.06.57336347 - Daniela Visaggio, daniela.visaggio@uniroma3.it; Department of Science, Roma Tre University, Viale Guglielmo Marconi 446, 00146 Rome, Italy; Phone: +39.06.57336408.

Table of contents

Table S1	Characteristics of bacterial strains used in this study	ii
Table S2	Iron concentration in culture media used for Ga(III) susceptibility testing	iii
Figure S1	Growth of reference non-fastidious CF pathogens in CAMHB, ID-CAMHB, ASM and RPMI-HS-CAA	iv
References		v

Table S1. Characteristics of bacterial strains used in this study[a]

Bacterial species	Strain	Country	Year	Source	Resistance	Reference
Achromobacter xylosoxidans	ATCC 27061^T	Japan	ns	ear discharge	ns	[1]
A. xylosoxidans	CF-2	Italy	2008-2010	respiratory secretion of CF patient	MDR	[2]
A. xylosoxidans	CF-3	Italy	2008-2010	respiratory secretion of CF patient	MDR	[2]
A. xylosoxidans	CF-4	Italy	2008-2010	respiratory secretion of CF patient	MDR	[2]
Burkholderia cenocepacia	LMG 16656^T	UK	1989	sputum of CF patient	ns	[3]
B. cenocepacia	FFC 0076	UK	ns	CF patient	ns	This study[b]
Burkholderia dolosa	LMG 18943^T	USA	ns	sputum of CF patient	ns	[4]
B. dolosa	FFC0305	USA	ns	CF patient	ns	This study[b]
Burkholderia multivorans	LMG 31010^T	Belgium	1992	CF patient	ns	[5]b
B. multivorans	454	Czech Republic	2002	CF patient	ns	This study[b]
Haemophilus influenzae	ATCC 49247	USA	1984	sputum of a pneumonia patient	ns	ATCC
H. influenzae	ATCC 9833	USA	ns	CF of patient with meningitis	ns	ATCC
H. influenzae	FC 89	Italy	2004-2009	CF patient	AMP, IPM	[6]
H. influenzae	FC 104	Italy	2004-2009	CF patient	ns	[6]
Mycobacterium abscessus	ATCC 19977^T	USA	1953	Knee infection	MDR	[7]
M. abscessus	ISS6	Italy	ns	unknown	MDR	This study[b]
M. abscessus	ISS7	Italy	ns	unknown	MDR	This study[b]
M. abscessus	ISS9	Italy	ns	unknown	MDR	This study[b]
Pseudomonas aeruginosa	ATCC 15692 (PAO1)	ns	ns	infected wound	ns	ATCC
P. aeruginosa	TR1	Italy	2009	respiratory secretion of CF patient	ns	[8]
P. aeruginosa	FM12	Italy	ns	respiratory secretion of CF patient	ns	[9]
P. aeruginosa	FM13	Italy	ns	respiratory secretion of CF patient	ns	[9]
Staphylococcus aureus	ATCC 25923	USA	1945	human clinical	ns	ATCC
S. aureus	BG-1	Italy	2016	CF patient	ns	This study[b]
S. aureus	BG-6	Italy	2016	CF patient	MRSA	This study[b]
S. aureus	BG-7	Italy	2016	CF patient	MRSA	This study[b]
Stenotrophomonas maltophilia	ATCC 13637^T	ns	ns	OP region of patient with mouth cancer	ns	ATCC
S. maltophilia	K279a	UK	ns	blood of non-CF patient	MDR	[10]
S. maltophilia	OBGTC23	Italy	2003-2005	sputum of CF patient	MDR	[11]
S. maltophilia	OBGTC26	Italy	2003-2005	sputum of CF patient	MDR	[11]
Streptococcus pneumoniae	ATCC 33400^T	ns	ns	unknown	ns	ATCC
S. pneumoniae	PFC-01	Italy	2010	CF patient	ns	[12]
S. pneumoniae	PFC-02	Italy	2010	CF patient	ns	[12]
S. pneumoniae	PFC-04	Italy	2010	CF patient	ns	[12]

[a] Abbreviations: ATCC, American type culture collection; AMP, ampicillin; CF, cystic fibrosis; CSF, cerebrospinal fluid; IPM, imipenem; MDR, multi-drug resistant; MRSA, methicillin-resistant *Staphylococcus aureus*; OP, Oropharyngeal; ns, not specified. ^{T} type strain.

[b] Identification of strains as member of *B. cepacian* complex was carried out by a triphasic analysis: (*i*) growth on the selective *Burkholderia cepacia* agar base (ThermoFisher); (ii) positive identification using the commercial system API 20NE (bioMérieux, Marcy l'Etoile, France); (iii) molecular identification by *recA*-PCR-Restriction Fragment Length Polymorphism as previously described[13], *M. abscessus* was isolated on Lowenstein-Jensen solid medium and identified by matrix-assisted laser desorption ionization-time of flight mass spectrometry (MALDI-TOF MS, Bruker Daltonics); *S. aureus* strains were identified by both manual (API-STAPH System; bioMérieux, Marcy-L'Etoile, France) and automate (VITEK 2 System, bioMérieux) biochemical test-based system

Table S2. Iron concentration in culture media used for Ga(III) susceptibility testing

Medium	Iron (μM)
CAMHB	3.305 ± 0.001
ID-CAMHB	0.143 ± 0.006
HTM	8.237 ± 0.008
DHTM	0.284 ± 0.001
THYB	17.136 ± 0.011
DTHYB	5.298 ± 0.002
ASM	3.880 ± 0.002
RPMI-CAA	0.210 ± 0.001
RPMI-HS-CAA	1.820 ± 0.002

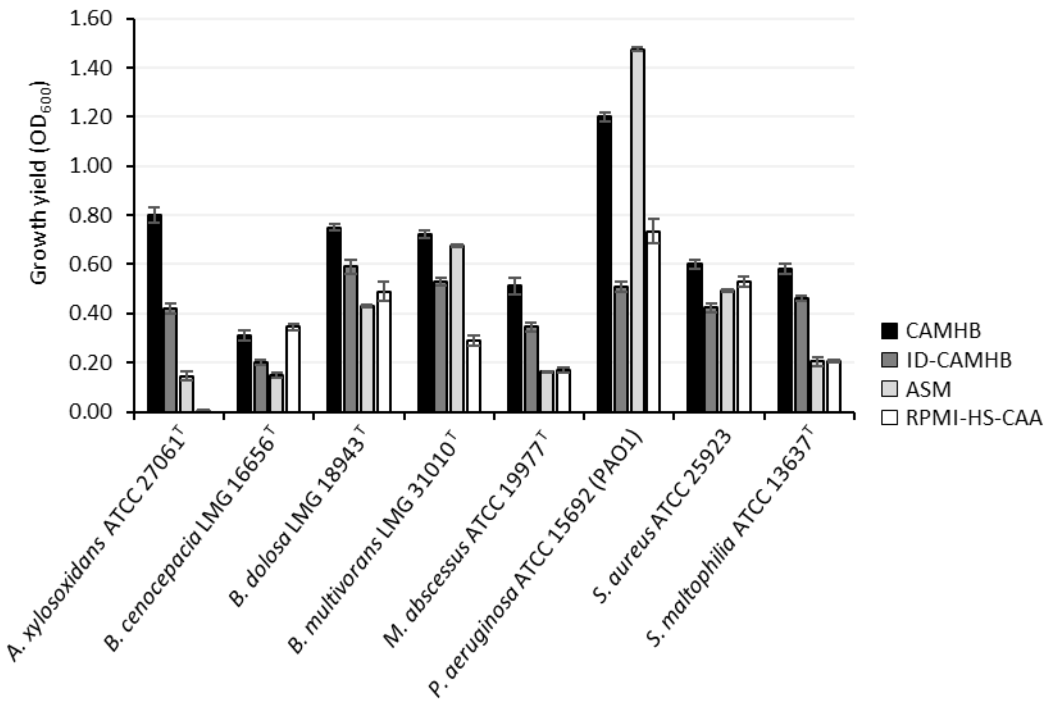

Figure S1. Growth of reference non-fastidious CF pathogens in CAMHB, ID-CAMHB, ASM and RPMI-HS-CAA. Individual bacterial strains were inoculated (*ca.* 5 x 10^5 CFU/ml) into 96-well microtiter plates containing CAMHB (black), ID-CAMHB (dark grey), ASM (light grey) and RPMI-HS-CAA (white). The OD_{600} was determined after 24-h incubation at 37 °C for all strains except *M. abscessus* ATCC 19977^T which required 72-h incubation. Data are the means of triplicate experiments ± standard deviation.

References

[1] Yabuuchi, E., Oyama, A. (1971) *Achromobacter xylosoxidans* n. sp. from human ear discharge. *Jpn. J. Microbiol. 15*, 477-481.DOI: 10.1111/j.1348-0421.1971.tb00607.x.

[2] Trancassini, M., Iebba, V., Citerà, N., Tuccio, V., Magni, A., Varesi, P., De Biase, R.V., Totino, V., Santangelo, F., Gagliardi, A., Schippa, S. (2014) Outbreak of *Achromobacter xylosoxidans* in an Italian Cystic fibrosis center: genome variability, biofilm production, antibiotic resistance, and motility in isolated strains. *Front. Microbiol. 5*, 138. DOI: 10.3389/fmicb.2014.00138.

[3] Govan, J.R., Brown, P.H., Maddison, J., Doherty, C.J., Nelson, J.W., Dodd, M., Greening, A.P., Webb, A.K. (1993) Evidence for transmission of *Pseudomonas cepacia* by social contact in cystic fibrosis. *Lancet 342*, 15-19. DOI: 10.1016/0140-6736(93)91881-l.

[4] Coenye, T., LiPuma, J.J., Henry, D., Hoste, B., Vandemeulebroecke, K., Gillis, M., Speert, D.P., Vandamme, P. (2001) *Burkholderia cepacia* genomovar VI, a new member of the *Burkholderia cepacia* complex isolated from cystic fibrosis patients. *Int. J. Syst. Evol. Microbiol. 51*, 271-279. DOI: 10.1099/00207713-51-2-271.

[5] Vandamme, P., Holmes, B., Vancanneyt, M., Coenye, T., Hoste, B., Coopman, R., Revets, H., Lauwers, S., Gillis, M., Kersters, K., Govan, J.R. (1997) Occurrence of multiple genomovars of *Burkholderia cepacia* in cystic fibrosis patients and proposal of *Burkholderia multivorans* sp. nov. *Int. J. Syst. Bacteriol. 47*, 1188-1200. DOI: 10.1099/00207713-47-4-1188.

[6] Cardines, R., Giufrè, M., Pompilio, A., Fiscarelli, E., Ricciotti, G., Di Bonaventura, G., Cerquetti, M. (2012) *Haemophilus influenzae* in children with cystic fibrosis: antimicrobial susceptibility, molecular epidemiology, distribution of adhesins and biofilm formation. *Int. J. Med. Microbiol. 302*, 45-52. DOI: 10.1016/j.ijmm.2011.08.003.

[7] Moore, M., Frerichs, J.B. (1953) An unusual acid-fast infection of the knee with subcutaneous, abscess-like lesions of the gluteal region; report of a case with a study of the organism, *Mycobacterium abscessus*, n. sp. *J. Invest. Dermatol. 20*, 133-69. DOI: 10.1038/jid.1953.18.

[8] Bragonzi, A., Paroni, M., Nonis, A., Cramer, N., Montanari, S., Rejman, J., Di Serio, C., Döring, G., Tümmler, B. (2009) *Pseudomonas aeruginosa* microevolution during cystic fibrosis lung infection establishes clones with adapted virulence. *Am. J. Respir. Crit. Care. Med. 180*, 138-145. DOI: 10.1164/rccm.200812-1943OC.

[9] Massai, F., Imperi, F., Quattrucci, S., Zennaro, E., Visca, P., Leoni, L. (2011) A multitask biosensor for micro-volumetric detection of N-3-oxo-dodecanoyl-homoserine lactone quorum sensing signal. *Biosens. Bioelectron. 26*, 3444-3449. DOI: 10.1016/j.bios.2011.01.022.

[10] Avison, M.B., Higgings, C.S., von Heldreich, C.J., Bennett, P.M., Walsh, T.R. (2001) Plasmid location and molecular heterogeneity of the L1 and L2 β-lactamase genes of *Stenotrophomonas maltophilia*. *Antimicrob. Agents Chemother. 45*, 413-419. DOI: 10.1128/AAC.45.2.413-419.2001.

[11] Di Bonaventura, G., Prosseda, G., Del Chierico, F., Cannavacciuolo, S., Cipriani, P., Petrucca, A., Superti, F., Ammendolia, M.G., Concato, C., Fiscarelli, E., Casalino, M., Piccolomini, R., Nicoletti, M., Colonna, B. (2007) Molecular characterization of virulence determinants of *Stenotrophomonas maltophilia* strains isolated from patients affected by cystic fibrosis. *Int. J. Immunopathol. Pharmacol. 20*, 529-537. DOI: 10.1177/039463200702000311.

[12] Pimentel de Araujo, F., D'Ambrosio, F., Camilli, R., Fiscarelli, E., Di Bonaventura, G., Baldassarri, L., Visca, P., Pantosti, A., Gherardi, G. (2014) Characterization of *Streptococcus pneumoniae* clones from paediatric patients with cystic fibrosis. *J. Med. Microbiol. 63*, 1704-1715. DOI: 10.1099/jmm.0.072199-0.

[13] Mahenthiralingam, E., Bischof, J., Byrne, S.K., Radomski, C., Davies, J.E., Av-Gay, Y., Vandamme, P. (2000) DNA-Based diagnostic approaches for identification of *Burkholderia cepacia* complex, *Burkholderia vietnamiensis, Burkholderia multivorans, Burkholderia stabilis,* and *Burkholderia cepacia* genomovars I and III. *J. Clin. Microbiol. 38*, 3165-3173. DOI: 10.1128/JCM.38.9.3165-3173.2000.
